# Editorial: Pre- or Post- School Influences on Learning Adaptations, Risks and Disabilities in Children and Adolescents: Overlapping Challenges for Public Health, Education and Development

**DOI:** 10.3389/fpubh.2021.651179

**Published:** 2021-03-31

**Authors:** Amedeo D'Angiulli, Nicole Letourneau, Eric R. Hamilton, Kimberly Schonert-Reichl, Gerry Leisman

**Affiliations:** ^1^Department of Neuroscience, Carleton University, Ottawa, ON, Canada; ^2^Faculty of Nursing, University of Calgary, Calgary, AB, Canada; ^3^Graduate School of Education and Psychology, Pepperdine University, Malibu, CA, United States; ^4^Department of Educational & Counselling Psychology, and Special Education, University of British Columbia, Vancouver, BC, Canada; ^5^Faculty of Social Welfare and Health Sciences, University of Haifa, Haifa, Israel

**Keywords:** children's health, learning adaptations, disabilities, risk and resilience, public health, prevention and intervention

The study of child and adolescent learning has generally focused on aspects specifically tied to individual academic performance. However, a new emerging perspective is that any “deficit” and/or disability and conversely any achievement is not the result of a single event, such as an isolated reaction, but it is formed, through numerous biosocial contributing variables, during a child's attempt to adapt to learning conditions and settings. The fit between such adaptations and normative criteria (set by educational and social standards) is often associated with labels such as “fulfillment,” “strengths” “resilience” or “weaknesses,” “risk,” “vulnerability” and “disability.”

This Research Topic explored the overlapping challenges and themes related to developmental adaptations (as defined above) in the context of formal and informal settings for learning primarily within childhood and adolescence.

To start off in this path, Jefferies et al. investigated the relationships between the multidimensional constructs of physical literacy and resilience in 227 school Canadian children aged 9–12 years old. They found that resilience was predicted by movement capacity, confidence, and competence, environmental engagement, and overall perceptions of physical literacy. Their research highlights the importance of introducing physical literacy in schools.

In a similar vein, Ato et al. examined the impact of temperament on academic achievement and sociometric status in a sample of 295 6–7-year-old Spanish children. Parents completed the Temperament in Middle Childhood Questionnaire, while sociometric status and academic achievement were derived from teachers' reports. Latent profile analysis showed that Children in the “Negative/Undercontrolled” profile were at higher risk for academic failure and peer rejection, while “Sociable/High regulated” showed the reverse pattern. The findings have very important (so far underexplored) implications for ways in which schools could integrate “difficult children.”

Two contributions focused on early developmental determinants. Firstly, Yao et al. examined the association of dopamine-related genes with mental and motor development and gene-environment interaction in 201 preterm and 111 term Taiwanese children, who were followed from 6 to 36 months and were genotyped for 15 single-nucleotide polymorphisms (SNPs) in dopamine-related genes (DRD2, DRD3, DAT1, COMT, and MAOA). MAOA SNPs were robustly associated with the mental (but not motor) development scores throughout early childhood in the premature children but not in the term counterparts. This warrants further investigations on whether the MAOA variants could help develop personalized interventions for preterm children.

Considering another early developmental period, Li et al. examined whether *executive function training* (EFT) could improve children's emotional competence (EC). Fifty-five 4-year-old Chinese children were assigned to either EFT or no-EFT group. Pre-test vs. post-test training between-and- within-subjects effects were analyzed to quantify improvement. EFT was associated with significantly higher scores on EC and changes in inhibition control and working memory abilities significantly predicted variation in EC. The findings suggest that intervening on inhibition control and working memory abilities via training may improve preschool children's emotional abilities.

At the other end of the spectrum, adult studies have shown that the concurrency of a smartphone-related task and walking can increase instability and risk of injuries. Caramia et al. recruited 29 young Italian adolescents to test whether walking with a smartphone increased fall and injuries risk, and to quantify these possible outcomes, participants were asked to walk along a walkway, with and without a concurrent writing task on a smartphone. Concurrency of walking and smartphone use resulted in reduced step length, gait speed and general aspects of gait stability, regardless of experience or frequency of use, suggesting that using the smartphone while walking may determine an increased risk of injury or falls also for young digital natives.

Adopting an approach to prevent air pollution, Zhao et al. developed the Brief Haze Weather Health Protection Behavior Assessment Scale-Adolescent Version (BHWHPBAS-AV), and tested its validity and reliability in two randomly selected districts of Baoding, China, and involving 22 middle-school classrooms and 1,025 valid questionnaires. The BHWHPBAS-AV scale showed promising reliability and validity suggesting it may be applied to assess adolescent haze weather health protection behavior, and help school and medical staff administer targeted behavioral and preventative interventions or health education programs.

In a second prevention study, Grant et al. assessed the cognitive profiles of 360 Canadian adults and children ranging from 7 to 80 years of age with disability in reading alone, mathematics alone and both (comorbidity), with tests widely used in both psychoeducational and neuropsychological batteries. Through a systematic exhaustive review of clinical neuroimaging literature, they mapped the complex set of domain-specific and domain-general impairments shown in the comorbidity of reading and mathematical disabilities to correspondingly plausible neuroanatomical substrates of dyslexia and dyscalculia. According to their hypothetical model, reading-math comorbidity seems due to atypical development of the left angular gyrus. This neuroeducational framework may assist to improve both early prediction and intervention across developmental periods.

Finally, Malboeuf-Hurtubise et al. reported a pilot study based on a new intervention, which combines mindfulness meditation and Philosophy for Children (P4C) activities, with the goal of improving mental health in pre-kindergarten children. Thirty-eight pre-kindergarten Canadian children took part in this study and were randomly allocated to the experimental or wait-list control conditions. Teachers completed pre- and post-intervention questionnaires. Although there were no significant effects, some improvement trends were found for internalized symptoms and hyperactivity. The results partly contradict previous research and suggest that mindfulness and P4C may not be effective intervention for mental health in children. However, the study also suggests a host of other confounding variables that might be responsible for the null findings and should be addressed in future research.

In summary, this Research Topic collection explored how children's preferences, profiles and predispositions are shaped by the social and biological activities that form the background of their everyday living. These papers addressed integrated multidisciplinary issues of education, development and public health, contributing practical examples of viable and sustainable local targeted programs, which, hopefully, may stimulate future research.

## Author Contributions

AD'A wrote the first draft. All authors contributed equally to edit to final version.

## Conflict of Interest

The authors declare that the research was conducted in the absence of any commercial or financial relationships that could be construed as a potential conflict of interest.

